# Clean Indoor Air in El Paso, Texas: A Case Study

**Published:** 2004-12-15

**Authors:** Monica H Eischen, Jennifer H Reynolds, Robin L Hobart, Ayala Patricia

**Affiliations:** Office on Smoking and Health, Centers for Disease Control and Prevention (CDC); Office on Smoking and Health, CDC, Amherst, Mass; Denver, Colo; El Paso, Tex

## Abstract

**Background:**

Exposure to secondhand smoke is an important preventable cause of illness and death. A Smoke-Free Paso del Norte Coalition in El Paso, Texas, led a drive to introduce an ordinance to protect nonsmoking persons from the health effects of secondhand smoke in public places. The ordinance was introduced in April 2001 and was passed on June 26, 2001.

**Context:**

El Paso is the fifth largest city in Texas and the largest border city in the United States. It is the 10th poorest city in the United States; 37% of its residents do not have health insurance. Seventy-eight percent of El Paso's residents are Hispanic/Latino. A large percentage of El Paso's restaurant and bar workers are recent immigrants from Mexico.

**Methods:**

Campaign activities included a letter-writing campaign to the *El Paso Times*, petition gathering, community outreach education, meetings with city council members, print and television advertising, a proactive media advocacy campaign, and a youth rally.

**Consequences:**

One month after the ordinance went into effect, an opinion poll found solid support for the new ordinance. Another survey conducted in December 2002 also found a 22% decline in adult smoking, from 22.1% in 1996 to 17.3% at the time of the survey.

**Interpretation:**

The El Paso campaign is an example of a successful grassroots campaign. El Paso's campaign relied on direct organizing to identify, recruit, and mobilize supporters, and involved relatively little paid media or paid advocacy efforts. These lessons are transferable to other communities, and the El Paso coalition serves as a model for developing a diverse, representative coalition in a predominantly Mexican American community.

## Background

The health effects of secondhand smoke are well documented; exposure to secondhand smoke is an important preventable cause of illness and death (1-3). Smoking bans have been developed as a legal tool to limit smoking in workplaces and other public areas (4), and they are recommended by the Task Force on Community Preventive Services as an effective intervention for creating smoke-free environments (5).

In April 2000, A Smoke-Free Paso del Norte Coalition was founded with funding for a four-year comprehensive tobacco control project from the Paso del Norte Health Foundation. Its primary objective was to win approval for an ordinance to protect nonsmoking employees and patrons from the health effects of secondhand smoke in all workplaces and public places, including restaurants, bars, bingo facilities, and bowling alleys.

In El Paso, the process for passing a new ordinance begins with introduction of the ordinance to the city council, followed by a city council vote. An ordinance such as a smoking ban is proposed by the El Paso City-County Health and Environmental District. Health and Environmental District policies must be approved by vote of the city council. There are eight members of the El Paso City Council. The mayor votes if votes are tied.

In April 2001, the Health and Environmental District introduced the ordinance, and the El Paso City Council approved it by a vote of seven to one on June 26, 2001.

The introduction of the ordinance in 2001 represented the second attempt to pass a clean indoor air ordinance in El Paso. The first attempt began in November 1994, when the El Paso Tabaco/Smoke-Free Coalition presented a proposal to the El Paso City-County Health and Environmental District to strengthen the city smoking ordinance. Although the Health and Environmental District approved the proposal, this initial ordinance campaign failed in the city council. In March 1996, the city council tabled the proposed ordinance indefinitely.

## Context

With a population of more than 550,000, El Paso is the fifth largest city in Texas and the largest border city in the United States. It is the 10th poorest city in the United States; 37% of its residents do not have health insurance, the highest percentage of uninsured in the nation (6). Seventy-eight percent of El Paso's residents are Hispanic/Latino (7). The Fort Bliss Army base and the University of Texas at El Paso are two important institutions that play a large role in the life of the community. On the other side of the Rio Grande River is El Paso's sister city, Ciudad Juarez, the fifth largest city in Mexico.

El Paso has a large number of blue collar and hospitality workplaces — the types of workplaces least likely to voluntarily protect workers from secondhand smoke (8). A large percentage of El Paso's restaurant and bar workers are recent immigrants from Mexico who are unlikely to know about the dangers of secondhand smoke, and because of a perception that their options are limited, they are not likely to seek jobs elsewhere (9). These factors played an important role in deciding which kinds of provisions to include in the ordinance.

Although tobacco industry opposition to clean indoor ordinances is commonplace (10) — and El Paso was no exception to this opposition — coalition members shared a belief that El Paso's efforts may have been somewhat protected by the opposition's underestimation of the community's ability to organize effectively. This underestimation may have been made because of the area's primarily low-income Hispanic population and historically low voter turnout. Opposition was strongest from the restaurant and bar associations, although they turned down an offer of assistance from the tobacco industry in their efforts to fight the ordinance.

One factor that also may have positively influenced the community was the smoke-free workplace restaurant ordinance in Las Cruces, New Mexico, which is located 35 miles from El Paso. Passed in 1995, the Las Cruces ordinance created a supportive environment for smoke-free policies and was further solidified when Las Cruces strengthened its ordinance again in 1997. The Tobacco-Free Las Cruces Coalition provided invaluable assistance to the El Paso Coalition as it began its own campaign, offering advice on what to look out for, how to undertake a grassroots organizing effort, who to include as coalition members, and other key stakeholders with whom it should meet.

## Methods

When the coalition was first formed in April 2000, its original plan was to conduct a four-year campaign in two parts, with an anticipated introduction of an ordinance in 2003. The first part was to be a comprehensive community education campaign preceding introduction of the ordinance. The second part was to be a year-long grassroots mobilization and media advocacy campaign leading up to ordinance approval. This plan changed dramatically, however, when the Health and Environmental District decided to introduce the ordinance well in advance of the coalition's readiness to run a campaign.

The ordinance was introduced earlier than the coalition expected because in fall 2000, a health district board member questioned an effort that had begun in the mid-90s to create an ordinance but had never progressed to a vote. By law, when a city entity like the health district requests that an issue be considered, the issue must be presented to the city council within two weeks. When the coalition learned that the issue was under question and that introduction of the ordinance could be imminent, it appealed to the health district to slow the process so that both the health district and the coalition could work together most effectively toward their common goal of creating an ordinance. The health district agreed to delay the introduction, but it was not willing to wait until 2003. Instead, the ordinance's introduction was scheduled for spring 2001. Thus, the coalition learned in November 2000 that it had only six to eight months to implement what it had previously planned to implement over four years.

**Table:** 

**Highlights of Activities for Passing Clean Indoor Air Ordinance in El Paso, Texas, 2000–2004**	
**1996**	First attempt at clean indoor air ordinance fails in El Paso City Council.
**April 2000**	A Smoke-Free Paso del Norte Coalition is founded with mission to win approval for an ordinance to protect nonsmoking persons from the health effects of secondhand smoke in public places. Coalition plans to introduce ordinance in 2003.
**June 2000**	Coalition members attend the Centers for Disease Control and Prevention's (CDC's) Summer Institute course on clean indoor air.
**November 2000**	Coalition learns that ordinance will be introduced by the Health and Environmental District in spring 2001. Fifteen-member task force is recruited and organized with diverse city-wide representation. News media advocacy campaign launches with visit to *El Paso Times* editorial board.
**December 2000**	Research on clean indoor air ordinances, opposition to clean indoor air laws, and the harmful effects of secondhand smoke is gathered and synthesized into education materials for the news media, city council, and general public.
**March 2001**	Petition-gathering effort with a focus on the faith community takes place, and 7000 signatures are hand-delivered to each of eight city council members, plus the mayor. Letter-writing campaign to *El Paso Times* editors and city council members begins. Ordinance task force members attend an Americans for Nonsmokers' Rights "Back to Basics" ordinance training.
**April 2001**	District introduces an ordinance to protect nonsmoking persons from the health effects of secondhand smoke in public places. A 30-second educational message airs on television to raise public awareness of secondhand smoke. Coalition members participate in educational session on mobilizing communities, conducted by New Mexico Department of Health.
**June 2001**	The ordinance is passed by a city council vote of seven to one.
**December 2001**	Coalition distributes 18,000 educational packets, including required decals, to all businesses, public places, and worksites in El Paso. Members of law enforcement responsible for enforcing the provision are trained.
**January 2002**	The clean indoor air ordinance goes into effect.
**February 2002**	*El Paso Times* and ABC affiliate (KVIA) sponsor an opinion poll, which finds solid public support for the new ordinance (13).
**February 2004**	The CDC and Texas Department of Health analysis shows that no statistically significant changes in restaurant and bar revenues occurred after the smoking ban took effect (15).

In November 2000, the coalition quickly formed a Clean Indoor Air Ordinance Task Force of 15 members to serve as the core team responsible for developing a campaign plan and directing day-to-day campaign operations. The coalition recruited key community leaders to join the task force. In addition to the voluntary health agencies, key supporters included Community Voices Tobacco Control Program (a project funded by the W.K. Kellogg Foundation and the American Legacy Foundation), state and local health departments, local law enforcement, local hospitals and community clinics, Planned Parenthood, the Independent School District and the Region 19 Education Center, a coalition of 18 churches, faculty from the University Health Sciences Center, a waiter/bartender, and a supportive (behind-the-scenes) local restaurant.

Task force members made community presentations to educate the public and recruit new supporters, and members identified a strong champion on the city council. The task force also developed a youth smoke-free coalition, whose efforts were deemed vital to the success of the ordinance. Task force members recruited youth volunteers through independent school districts.

The El Paso coalition relied on models developed and published by Americans for Nonsmokers' Rights (ANR) in *Clearing the Air: Citizen Action Guide* (11) and by the Centers for Disease Control and Prevention (CDC) in *Best Practices for Comprehensive Tobacco Control* (12) to develop a clean indoor air campaign. In 2000, members of the coalition attended the CDC's Summer Institute course on clean indoor air. This week-long course provided detailed information and skill-building sessions focused on grassroots organizing, coalition building, and other important elements of developing a clean indoor air campaign.

The coalition also sought information and technical assistance from ANR, the CDC, the Texas State Department of Health, the Tobacco-Free Las Cruces Coalition, voluntary health agencies, and other local coalitions across the country with experience passing local smoke-free ordinances. In March 2001, ordinance task force members attended an ANR "Back to Basics" ordinance training, and in April 2001, a member of the New Mexico Department of Health conducted training on mobilizing communities.

One of the key issues debated by coalition members early in the campaign was which provisions to include in the ordinance. After much discussion, the coalition decided to draft a comprehensive ordinance creating full protection from secondhand smoke in all workplaces, including free-standing bars. Although including free-standing bars was a radical idea for its time, the coalition considered it vital to promote a comprehensive workplace ordinance. The coalition countered criticism of the free-standing bar provisions by emphasizing the message that secondhand smoke is a health hazard that affects all workers, and all workers deserve protection. The coalition also pointed out that a comprehensive ordinance creates a level playing field; exempting some workplaces but not others might offer an unfair competitive advantage to the free-standing bars over restaurants and bars attached to restaurants.

### Public awareness campaign

**Figure:**
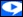
Watch KFOX news coverage in English (rm 3.6mb)Transcripts of this clip in English and Spanish are also available. You will need RealOne Player to view these videos. Learn more about RealPlayer.

**English Transcription of KFOX News Clip**Meanwhile El Paso Mayor Ray Caballero is celebrating the one-year mark of our own straight smoking ban. Caballero unveiled new numbers out just today that show not only our local restaurants and bars unaffected by the ban but they made more profit than ever before. The year-end sales tax receipts show El Paso's bars and restaurants made about 1.7 billion dollars last year compared to 1.6 billion in 2001. Mayor pro tem Larry Medina says, the smoking ban not only makes El Paso more money, it saves lives: “We are not talking about one life or a dozen lives or just a couple of hundred lives. We are literally talking ladies and gentlemen over the years to come literally thousands of people’s lives we are saving just here in little old El Paso in little old Texas.”Medina says representatives from cities across the country are now using El Paso as a model for their own non-smoking ordinances.

**Figure:** Vea la cobertura de noticias por Canal 26 Noticias en Español (rm 4mb)

**Spanish Translation of KFOX News Clip**Mientras tanto, el Alcalde de El Paso Ray Caballero está celebrando el primer aniversario de nuestra propia iniciativa, la prohibición categórica de fumar. Caballero dio a conocer los recientes números que se conocieron justamente hoy día los cuales demuestran que no solamente nuestros bares y restaurantes no se han visto afectados por la prohibición sino que tuvieron mayores ganancias que antes. Los recibos de los impuestos sobre las ventas de fin de año demuestran que los bares y restaurantes de El Paso llegaron a la cifra de 1,7 mil millones de dólares durante el año pasado comparada a la cifra de 1,6 mil millones en el año 2001. El Alcalde interino Larry Medina dice que la prohibición de fumar no solamente crea más ingresos, sino que salva vidas: “No estamos hablando de una vida o de una docena de vidas ni siquiera de algunos cientos de vidas. Hablamos literalmente señoras y señores, que en el transcurso de los próximos años salvaremos literalmente miles de vidas aquí solamente en nuestro querido El Paso en nuestro querido estado de Texas.”Medina dice que los representantes de las ciudades a través del país se utilizan ahora como un modelo a El Paso para sus propias ordenanzas de no fumar.

One of the first steps taken by coalition members was to meet with members of the *El Paso Times* editorial board to counter some negative publicity that had arisen earlier in the year. The *El Paso Times* is a regional newspaper with a daily readership of approximately 250,000 that serves residents of far West Texas and southern New Mexico.

During December 2000, the coalition focused on gathering research on various kinds of clean indoor air ordinances, opposition to such ordinances, and the harmful effects of secondhand smoke to prepare educational materials for the news media, city council, and El Paso citizens.

During March 2000, a task force member active in the faith community led a petition-gathering effort among 18 churches and collected 7000 signatures that were hand-delivered to each of eight city representatives, plus the mayor.

The coalition worked with an advertising agency to produce an educational television message on secondhand smoke for $2000; it cost $8000 to air the ad during April. TRUST for a Smoke-Free Texas provided a grant of $2500 to support placement of a print advertisement, which was developed on a pro bono basis by the ad agency and placed in the *El Paso Times* shortly before the city council vote. The Texas Division of the American Cancer Society provided $5000 to support printing (e.g., fact sheets, buttons) and postage costs.

On the day of the vote, a large youth rally took place at city hall, and three young people — a nine-year-old boy and two girls from a local high school — delivered testimony to city council members on the health effects of secondhand smoke. The coalition gave out buttons with the words "I support Clean Indoor Air" and flashing lights to all those in attendance. The event was packed with citizens and members of the news media.

After the ordinance passed, the coalition worked with the Health and Environmental District to develop an educational packet for 18,000 El Paso businesses. The packet included a detailed explanation of the ordinance and its provisions, sample employee policies, and decals, which were required to be placed on business entrances. In addition, the coalition held training sessions for members of law enforcement who were responsible for enforcing the ordinance.

## Consequences

The ordinance went into effect in January 2002. In February 2002, the *El Paso Times* and the ABC affiliate (KVIA) sponsored an opinion poll. The poll found solid support for the new ordinance; 93% indicated they would go out to restaurants and bars as often (49%) or more often (44%) as a result of the ordinance (13).

In December 2002, 11 months after the ordinance went into effect, the Paso del Norte Health Foundation sponsored a household telephone survey, which also found strong support for the ordinance; after a full year of implementation, 78.5% indicated they supported the ordinance, and only 10.9% opposed it (the rest reported no opinion). Although general knowledge about the existence of the ordinance was high, familiarity with the ordinance's specifics was not. The Paso del Norte Health Foundation survey also found a 22% decline in adult smoking, from 22.1% in 1996 to 17.3% at the time of the survey in 2002 (14). An economic impact analysis by the mayor found that total sales subject to state sales tax in eating and drinking establishments continued to grow at a steady pace after the ordinance went into effect (14). Total sales for the first two quarters of 2002 increased by 4.4%, up slightly from the prior year's increase of 2.5% (14). The number of employed waiters and waitresses also increased by 300 from 2001 to 2002 (14). The Texas Department of Health and the CDC subsequently analyzed sales tax and mixed-beverage tax data during the 12 years preceding and one year after the smoking ban to further assess whether the El Paso smoking ban affected restaurant and bar revenues; it was determined that no statistically significant changes in restaurant and bar revenues occurred after the smoking ban took effect (15).

Following enactment of the ordinance, the local restaurant association seemed resigned to complying with the new ordinance, but local bars continued to oppose it, attempting to collect enough signatures to force the ordinance to a referendum. The coalition closely tracked this effort, which failed to collect enough valid signatures to qualify (in part because the petitions did not use uniform language). Opponents also attempted to place the ordinance back on the city agenda for discussion. Coalition members monitored the council agenda and sent representatives when the ordinance was listed as an agenda item. After opponents twice failed to attend, the city council stopped putting the issue on the agenda.

## Interpretation

This campaign was a collaborative, unified effort, based in and owned by the community. Several components led to its success. One, the coalition consistently and uncompromisingly focused on the issue of worker's health and protection. Although some coalition members opposed including bars in the ordinance, the coalition stayed consistent with the message that bar workers deserved protection as much as workers in other workplaces.

Two, the coalition members sought training on policy and media advocacy. The coalition received technical assistance and support from ANR, the CDC, the voluntary health agencies, and from other local coalitions with experience working on smoke-free ordinances (in particular, the Tobacco-Free Las Cruces Coalition).

Three, the coalition was diverse, drawing from many sections of the community (e.g., health groups, law enforcement, educational groups, church groups, public agencies).

Four, the coalition recruited and developed youth leaders and empowered the youth coalition to set its own goals for the ordinance campaign. The youth were vital to the letter-writing campaign and held a rally the day of the council vote; their testimony at the public hearing was extremely persuasive to city council members.

Five, the coalition found and cultivated a strong champion on the city council. This champion was passionate and enthusiastic in his support for the ordinance, stayed in close communication with the coalition about developments and strategy, and brokered no compromises.

Six, the coalition was proactive with the news media. Members developed and distributed key speaking points to committed activists, provided the news media with background information, facts, and statistics, and monitored news media coverage (responding immediately to any negative coverage).

The El Paso campaign is an example of a grassroots campaign. It relied on direct organizing to identify, recruit, and mobilize supporters and involved relatively little paid media or paid advocacy efforts. The broad lessons from this campaign are transferable to other communities, especially as the issue of clean air for a worker's health and protection grows (16). In addition, the El Paso Coalition serves as a model for developing a diverse, representative coalition in a predominantly Mexican American community.

One caution to coalitions considering the El Paso initiative is that the time frame to educate the community and organize grassroots support was considerably compressed because of factors outside the coalition's control. Ideally, coalitions will have more time to educate the public and decision makers and recruit and mobilize grassroots supporters. However, it is also true that if too much time is allotted for the education and mobilization phase, coalition members can lose focus and energy and have a more difficult time putting aside other responsibilities. Therefore, coalitions must gauge an appropriate period that takes into account all of these issues.
